# Uptake and Scalability of a Peritoneal Dialysis Virtual Care Solution: Qualitative Study

**DOI:** 10.2196/humanfactors.9720

**Published:** 2019-04-16

**Authors:** Lianne Jeffs, Trevor Jamieson, Marianne Saragosa, Geetha Mukerji, Arsh K Jain, Rachel Man, Laura Desveaux, James Shaw, Payal Agarwal, Jennifer M Hensel, Maria Maione, Nike Onabajo, Megan Nguyen, R Bhatia

**Affiliations:** 1 Lunenfeld-Tanenbaum Research Institute Sinai Health System Toronto, ON Canada; 2 Institute of Health Policy Management and Evaluation University of Toronto Toronto, ON Canada; 3 St. Michael's Hospital Toronto, ON Canada; 4 Institute for Health System Solutions and Virtual Care Women's College Hospital Toronto, ON Canada; 5 London Health Sciences Centre London, ON Canada; 6 Department of Family and Community Medicine University of Toronto Toronto, ON Canada; 7 St. Michael's Hospital Toronto ON, ON Canada

**Keywords:** virtual care solutions, peritoneal dialysis, qualitative research, patient-centric care, chronic kidney disease

## Abstract

**Background:**

Early research in the area of virtual care solutions with peritoneal dialysis (PD) patients has focused on evaluating the outcomes and impact of these solutions. There has been less attention focused on understanding the factors influencing the uptake, usability, and scalability of virtual care for chronic kidney disease (CKD) patients receiving PD at home.

**Objective:**

In this context, a study was undertaken to (1) assess and understand the factors influencing the uptake of a virtual care solution and (2) provide recommendations for the scalability of a virtual care solution aimed at enhancing CKD patients’ outcomes and experiences.

**Methods:**

This study used a qualitative design with semistructured interviews and a thematic analysis approach. A total of 25 stakeholders—6 patients and 3 caregivers, 6 health care providers, 2 vendors, and 8 health system decision makers—participated in this study.

**Results:**

The following three primary mechanisms emerged to influence the usability of the virtual care solution: (1) receiving hands-on training and ongoing communication from a supportive team, (2) adapting to meet user needs and embedding them into workflow, and (3) being influenced by patient and caregiver characteristics. Further, two overarching recommendations were developed for considerations around scalability: (1) co-design locally, embed into the daily workflow, and deploy over time and (2) share the benefits and build the case.

**Conclusions:**

Study findings can be used by key stakeholders in their future efforts to enhance the implementation, uptake, and scalability of virtual care solutions for CKD and managing PD at home.

## Introduction

End-stage renal disease (ESRD) continues to prevail as a global public health problem affecting over 700,000 Americans [1] and more than 41,000 Canadians [2]. ESRD and chronic kidney disease (CKD) are frequently associated with substantial health care and societal costs [3,4]. People with CKD experience challenges accessing kidney care due to limited health care resources, which is exacerbated by geographical barriers for those patients who live in remote or underserved areas [5-8]. In this context, patients and their family members must travel long distances to obtain care, which adds stress, imposes additional costs, and contributes to the poorer quality of care [9] and, in some situations, mortality [10]. Barriers to access kidney care and associated costs may worsen as the prevalence of CKD increases with the rising aging population, who may experience multiple comorbidities that will require multiple medications to treat [4,9]. Alternate CKD care delivery models are needed to address these challenges faced by CKD patients to ensure more flexible, convenient, person-centered care delivery models, particularly for those living in rural and remote locations [11].

Peritoneal dialysis (PD) is one strategy to mitigate challenges with accessing care for managing CKD, particularly ESRD. Globally, 190,000 patients are receiving daily PD at home [12] following education and training by a specialized dialysis health care professional team [13,14]. Outcomes associated with PD include increased satisfaction [13], increased quality of life [15,16], and survival advantage, especially in the first few years of therapy [17-19], with decreased costs to the health care system [20]. Virtual care, often referred to as telehealth or telemedicine, is a rapidly evolving area where health care services are delivered, in part, through technology, including clinician-to-clinician, clinician-to-patient, and patient-to-mobile health technology communication that aims to enhance patients’ self-management of their disease in a home setting [4,17,20,21]. Technology includes remote monitoring [4,21,22], mobile phones [5,23], virtual wards [24], and Web-based eHealth portals [25]. Virtual care, coupled with PD, has the potential to improve access for CKD care for patients in their own homes [26-30].

A recent systematic review reported a significant decrease in hospital readmission, emergency room visits, number of days in the hospital, and increased compliance with home self-management programs associated with the use of virtual care solutions in the CKD population [4]. To date, research in this area has focused on evaluating the outcomes and impact of these solutions. There has been less attention focused on the factors that lead to the success or failure in uptake and scalability of telehealth programs for the CKD population [21]. Gaining insight into this gap may yield useful knowledge in improving the uptake and scalability of the virtual care solution (ie, telehealth) that can be used to improve future implementation efforts and ultimately improve patient and health service utilization outcomes. In this context, as part of a larger study, the qualitative arm included an exploration of the factors influencing the uptake of, and recommendations for, scalability of a virtual care solution aimed at enhancing CKD patients’ outcomes and experiences.

## Methods

### Study Design

This qualitative study design was employed as part of a larger evaluation of the eQ Connect (eQOL Inc) intervention that includes a parallel-arm, randomized controlled trial (RCT). The aim of the RCT, CONNECT Trial, is to determine if utilizing a virtual care solution that includes remote monitoring software, eQ Connect, improves selected clinical outcomes for PD patients. The study protocol provides more detail of the RCT [31]. Ethics approval for the qualitative study design was obtained from the Research Ethics Boards at Women’s College Hospital, St Michael’s Hospital, London Health Sciences Centre, and Humber River Regional Hospital, all located in Ontario, Canada.

### Study Setting

Participants were recruited from two hospitals: one urban teaching hospital, London Health Sciences Centre, and one community hospital, Humber River Hospital, from Ontario, Canada. Collectively, over 200 PD patients receive care from these two centers. Eligible patients were approached during their regularly scheduled clinic visit at the PD clinics.

### Theoretical Framing

The theoretical framing of the qualitative design included an integration of the following three key frameworks: (1) an adaptation of the Reach, Effectiveness, Adoption, Implementation, and Maintenance (RE-AIM) framework [32]; (2) Institute for Healthcare Improvement’s Triple Aim [33]; and (3) a scalability framework [34]. Specifically, we postulated that the successful uptake and scalability of the intervention would be influenced by a series of factors. These factors include leadership engagement and culture, communication methods, and social networks; structures including a learning system that incorporates training, support, and infrastructure that connects adopters and experts; and a data system to support measurement for improvement. Refer to the protocol for further details [31].

### Virtual Care Solution Description

The virtual care solution included a remote monitoring software, eQ Connect (eQOL Inc), that provides support for patients undergoing PD. eQOL is a Canadian health technology company based in Toronto, Ontario, specializing in the development and deployment of innovative solutions to enable patients to better manage their care outside of the hospital environment. The solution provides up-to-date patient information to the health care team and aims to promote patient adherence with PD regimens. The platform consists of a patient-facing interface, Patient Portal, that operates on a tablet (ie, iPad Mini 2 running Apple iOS); the platform is designed to record and upload data (eg, treatment progress, health status, and supply usage) easily and securely over the Internet to a secure data center. Information is transferred to a compliant secure data center where clinicians can gain access to the data by logging in to the Support Portal. A more detailed description of eQ Connect is provided in a published protocol paper [31].

### Data Collection and Analysis

The qualitative component included semistructured interviews with the principal stakeholders involved in the implementation of the eQ Connect app process: patients and caregivers, health care providers, and health system decision makers. Qualitative interviews included questions about (1) participants’ experiences of learning about and using the technology; (2) changes to health care provider workflow required to use the technology effectively; (3) organizational changes needed to support the technology; and (4) health system barriers and facilitators to effective implementation, evaluation, and scalability. Interviews were conducted through a telephone conversation at a time convenient to study participants between baseline and 3 months of implementation of eQ Connect from March to June 2017. The average length of time for interviews with stakeholders was as follows: patients and caregivers, 25 minutes (range 11-44 minutes); health care providers and vendors, 22 minutes (range 8-44 minutes); and health system decision makers, 49 minutes (range 44-59 minutes).

Qualitative interviews were conducted and recorded by experienced qualitative research assistants who then transcribed the interviews into Word documents, prepared the documents for qualitative analysis, and analyzed the interviews using thematic analysis [35]. Specifically, a coding schema was constructed and used to categorize the narrative text. This analytical process involved two researchers reviewing the transcripts line-by-line separately to identify sections of text that serve as codes. The researchers met to determine the codes and categories through consensus and the researchers developed themes and subthemes from the categorical data through consensus. As a final step to ensuring methodological rigor, the principal investigator reviewed all the original transcripts with the emergent coding schema. For more details on the study methods, refer to the published protocol [31].

## Results

### Sample Characteristics

Overall, the qualitative component involved 25 participants from the following stakeholder groups: 9 (36%) end users, including 6 (24%) patients and 3 (12%) family caregivers; 6 (24%) health care providers; 2 (8%) vendors; and 8 (32%) health system decision makers. In terms of the 9 patients and family caregivers, there were 5 (56%) males and 4 (44%) females. The average age of the patients and their caregivers was 66 years (range 41-86). Of the 9 patients and caregivers, 5 (56%) were married, 2 (22%) were common law, 1 (11%) was single, and 1 (11%) was divorced. Of the 9 patients and caregivers, 7 (78%) were educated at a university or college level and 2 (22%) at a high school level. The average length of time patients had been managing their CKD was 9.7 years (range 1-31). In terms of the 6 health care providers, 3 (50%) were project coordinators, 2 (33%) were nurses, and 1 (17%) was a physician. The vendor cohort of 2 participants included 1 (50%) product development manager and 1 (50%) clinical coordinator. No demographic information was obtained from the participating health system decision makers that drew from a variety of provincial agencies (eg, funder and networks).

### Themes

The following three factors associated with the uptake of eQ Connect by patients receiving PD at home emerged: (1) receiving hands-on training and ongoing communication from a supportive team, (2) adapting to meet user needs and embedding into workflow, and (3) being influenced by patient and caregiver characteristics. Further, the following two overarching recommendations emerged for considerations around scalability of eQ Connect: (1) co-design locally, embed into the daily routine and workflow, and deploy over time and (2) share the benefits and build the case.

### Influencing Factors

#### Receiving Hands-On Training and Ongoing Communication From a Supportive Team

This theme captured how patients and their caregivers valued the opportunity to receive a face-to-face, hands-on, brief training session on how to use the telemonitoring equipment and iPad as well as having access to, and receiving timely communication from, a supportive team (ie, health care providers in clinic and vendor). Patients described the face-to-face training session as “simple, short, and very good at orienting” them to using the tablet as part of their daily care routine. One caregiver noted the following:

...her teaching was good it was just a lot to take in in an hour. I was given all kinds of information the very first time the nurse talked to us and I came home and read it through and then when they called and said we were accepted [into the trial] that was fine.

Caregiver

Further, ongoing check-ins from the research and clinic staff ensured that the patients and caregivers, when present, knew how to use eQ Connect.

Patients also valued having access to the vendor if problems arose with the technology and being able to communicate directly with the staff at the CKD clinic, who they described as “very responsive.” One patient described the following:

They are always there, they always call me back—I’ve hardly had to call them. If I have a small problem, I’ll just message the support staff at the hospital and they will message me back an answer. They are always on top of it...right through the iPad.

Patient

Health care providers at the clinic also described the ability to connect with patients and their caregivers through the iPad, as illustrated in the following narrative:

It’s a great way to keep connected with the patient in the home and it’s also a positive thing because uh when you’re looking at the iPad, it’s a communication tool with the messaging center that can help address issues that patient might have without having to call us.

Health care provider

#### Adapting to Meet User Needs and Embedding Into Workflow

This theme reflects how the vendor adopted the virtual care solution to meet user—patients’ and providers’—needs and embedded them into the providers’ daily workflows. Patients, caregivers, and providers described how they would raise issues around technical glitches of eQ Connect and how the vendor would be receptive and adapt the functionality of the tablet to address the issues (eg, challenge entering data on the screen). This is noted in the following excerpt from a patient:

Whenever I did the incorrect inputting, I would be getting a phone call and they would touch base with me. I like the new way they’ve done the effluent screen—I told you that I kept incorrectly inputting, now it’s all on one screen you can see your three different things you have to input and I do like that. I like it when it’s all visual on one page. But that was certainly an improvement when they did that.

Patient

Another patient shared the following:

I gave her some ideas that I thought would make it easier and they seemed very receptive and then they [vendor] changed that.

Patient

The vendor also encouraged the health care team to “reach out and give feedback at any time.” The vendor further noted the following:

I work together with the team to customize the software specifically to different sites. We meet with the nurses and doctors and find [out] about their specific needs and the demographics of their patient population.

Vendor

The adaptability of the iPad by the vendor to meet patient and provider needs was also described by health care providers, as one health care provider noted:

That’s all evolved, they’ve either added more—the nurses were finding what wasn’t working and what the patients were finding that was more problematic that they took away and added and then they do the education of what has changed and how they need to change it.

Health care provider

The vendor also described how important it was to embed eQ Connect into health care providers’ daily workflows and patients’ daily routines; this was illustrated in this quote from a vendor:

The nurses have been very receptive because it is new and it’s a change; there are things that we have to work through with the nurses in terms of fitting it in into how they do things and their workflow, and also making sure everyone’s comfortable with the software.

Vendor

#### Being Influenced by Patient and Caregiver Characteristics

This theme emerged from the following subthemes: patients describing themselves as currently being stable and managing health issues, having tablet literacy or experiencing initial anxiety with eQ Connect, and being supported by a caregiver. Most patients described themselves as being stable at the moment and their condition as “hasn’t gotten worse, hasn’t gotten better,” which was reiterated by their health care providers. Some of the patients had shared that they have been dealing with CKD and its manifestations, alongside other conditions (eg, high blood pressure and diabetes), for years and attributed not improving to the progression of their CKD. Patients had varying levels of tablet literacy, with some having previous experience and established comfort, as described by one patient as “I wasn’t scared of using the iPad” and by another patient as “not a learning curve for me” and “I found it pretty simple to use”; others shared they had initial anxiety, but that with time and use they were able to use the tablet effectively.

This later finding was observed by the vendor who stated the following:

We find that some of our older patients have a little bit more time to get used to the technology, so for some of them it’s a matter of just using it repeatedly until they get comfortable.

Vendor

Health care providers also noted the comfort level of patients using the tablet effectively, as described in the following quote:

It depends on the patient’s aptitude with technology, on the demographic of the patient, and the patient population, because I think it is very easy to use but if the patient is a much older patient and obviously doesn’t speak English, then it’s kind of like one more thing the patient thinks, “Oh no, I don’t want to have to bother with this.”

Health care provider

Supportive caregivers were also identified as a factor in using the tablet and, in some cases, the caregiver was entering the data on the tablet, as stated by a patient in the following quote:

My wife is doing that iPad because I cannot see the numbers, she inputs and I never touch that iPad.

Patient

### Recommendations for Scalability

#### Codesign Locally, Embed Into Daily Routine and Workflow, and Deploy Over Time

Given how patients, caregivers, and providers valued the adaptability features of eQ Connect, future efforts to scale would benefit from a co-design approach. In our study, technological challenges (eg, entering data, small font and tablet size, and slow operating system) were resolved when brought to the attention of the vendor, who listened to feedback from the patients and health care providers. As one health system decision maker noted, future efforts “...need to think [about] the design approach, particularly how you set it up from the beginning to work the way it should for clinicians, data analysts, and patients using the app.” It is also imperative to integrate eQ Connect into existing workflows and care processes for health care providers and the daily life of patients and their caregivers, so that it is comfortable, convenient, and efficient to use. This is illustrated in a quote by a health care provider who shares, “It just needs to be woven into the fabric of their daily routine, because otherwise, usually there’s pushback when there’s just one more thing piled on top of their already hectic schedules.” Further, a phased-in deployment over time, particularly to support the smaller size of the vendor organization, to ensure that local adaptation and co-design can occur is also recommended, as noted by a vendor who stated, “You wouldn’t try to roll out to all different dialysis clinics across [the province]—it would be a continuing process, definitely be a gradual rollout adding patients and adding sites on to it.”

#### Share the Benefits and Build the Case

Another key recommendation for the scalability efforts of eQ Connect is to share the benefits of the pilot and build the case for ongoing investment for the virtual care solution. This includes communicating the key functionalities of the virtual care solution, such as real-time monitoring, surveillance, and communication; reminders for inventory management; and accessible technological support and clinical expertise by *word of mouth* (ie, informally) and through *networks* (eg, Ontario Renal Network and media network), highlighting the benefits and experiences shared by patients. As one health care provider stated, “It would be awesome to get patients’ input—there’s a lot of positive feedback that I’ve heard from patients. Sharing the benefits, experiences of patients and nurses.” Vendors and health system decision makers also described the importance of the need to strategically align the introduction of virtual care solutions, in this case eQ Connect, to broader health policy issues (ie, the economic, social, and health burden associated with CKD) and to key stakeholders’ priorities (eg, Local Health Integrated Network [LHIN] and Patients First, Ministry of Health and Long-Term Care platform). Part of building the case will be to leverage the passion and expertise of leaders (ie, having super users is suggested). Support from leadership at all levels (eg, health care team, health care organization, and government) will be required, as will ongoing rapid-cycle evaluations to inform how best to sustain gains met and spread to other patients and their caregivers. As one health system decision maker noted, “We are really looking to scale now, and if it’s not aligned with a LHIN priority or ministry priority, no one is going to care—so it’s wonderful work, but it has to be part of a broader strategy.”

## Discussion

### Principal Findings

This study delineated influencing factors and recommendations for scalability of a virtual care solution aimed at enhancing outcomes and experiences of CKD patients receiving PD at home. To our knowledge, this is one of the first empirical papers to elucidate factors that influence uptake and recommendations for scalability from multiple stakeholder groups.

Our study finding regarding how the hands-on training by the vendor is highly valuable is consistent with other literature that identifies using a hands-on learning approach as a key strategy for future usability of new technology [11]. In our study, the orientation provided to patients and caregivers was short in nature with minimal training that may reflect a well-designed product [36]. Similar to other studies using virtual care solutions [9,37,38], the ongoing accessibility and support from the CKD clinic team and vendor that patients had was key to using eQ Connect. Having virtual care solutions that are complimentary to, and integrated with, existing models of care is essential for usability and acceptance of new technology to support self-management and adherence [4,39]. In our study, the timely two-way exchange of data by patients, and in some cases their caregivers, with health care providers using eQ Connect made patients feel connected and supported. Trust between patients and health care providers and supportive communication between informal and professional caregivers are critical factors for the successful implementation of virtual care solutions for CKD and the provision of hemodialysis in the home environment [40,41].

Another prominent intervention mechanism, adapting the virtual care solution to meet the user’s end needs and the recommendation to co-design locally in scalability efforts, adds to the existing literature base on the critical role of engaging and meeting the needs of end users [36,42-45]. User involvement in the design and development process is a fundamental human factors design principle and offers many benefits. Specifically, when users are involved, devices are created and adapted to reflect what users’ needs are across their illness trajectories [36]. More recently, there are policy-level calls to include patients in identifying key virtual care strategies that will help the health system to be more patient-centric in nature [45]. Wider adoption of virtual care solutions requires processes to be redesigned from a patient-centric perspective to establish and sustain patient acceptance of care technology innovation [46]. In our study, there were variations around the health status of patients and tablet literacy that could have impacted the ongoing use and treatment adherence and were addressed over time by the CKD clinic team and vendor.

The co-design and adaptability of eQ Connect enabled technical and tablet literacy challenges to be addressed; they also enabled patients and their caregivers to continue to use the virtual care solution in their daily care routine and enabled an integration into the CKD clinic team’s daily workflow. This finding exemplifies another human factor design principle around having an intuitive design that is embedded into existing care practices of patients and workflow of health care professionals [11,36,45,46] that should be less complicated and time demanding [9]. Other good design elements that emerged in a recent qualitative study include aesthetic appearance, practicality and ease of use, and supportive platforms to enable flow of data [36].

In addition to co-designing locally using a patient-centric approach, our study further highlighted the need to deploy over time and ensure that the vendor has the capacity to scale up the virtual care solution. This has implications for scalability of user-centric designs that require tailoring to specific contextual situations. Reconciling the trade-offs around upfront investment in innovation and future benefit and cost-efficiencies will require sharing the benefits of, and building a case for, continued investment in eQ Connect as an effective, patient-centric virtual care solution for CKD patients receiving PD at home. Specifically, sharing the positive impact eQ Connect had on participants, particularly patients and their caregivers and health care professionals, is paramount, as are continued efforts to strategically align this work with broader health policy issues and key stakeholder priorities. Further research is required to examine and explore the outcomes and experiences using large-scale studies in different contexts associated with this patient-centric virtual care solution for CKD patients receiving PD at home.

### Limitations

Study findings need to be interpreted with the following limitations. Given the study was conducted at two hospitals in Southern Ontario, the transferability of the qualitative data to other types of health care organizations may be limited. Selection biases may also have existed, as participants volunteered to participate in the study. There was a small sample size with a limited number of patients enrolled at each site to recruit into the study. Finally, the interviews were conducted during a 3-month window after the initial training session. This may have resulted in varying levels of familiarity with the app at the time of the interview. It is important to note that we do not feel this would bias our results and that interviewing parties with varying experience is important to capture issues relating to early usage while mitigating the impact of the learning curve.

### Conclusions

Our study involved multiple stakeholder groups to elucidate the factors that influence uptake and recommendations for scalability of a virtual care solution for CKD patients receiving PD at home. Study findings can inform key stakeholders in their future efforts to enhance the uptake of implementation and scalability of virtual care solutions for CKD patients receiving PD at home that may also have relevance for other chronic diseases. Specific recommendations include the following: provide hands-on training and ongoing, timely support from the care team; co-design locally using a patient-centric approach adapting to meet user needs; embed into patients’ daily routines and health care professionals’ workflows; and deploy over time. Future efforts to scale up eQ Connect will require sharing the positive impact of eQ Connect and continued efforts to strategically align this work with broader health policy issues and key stakeholder priorities. Further research is required to examine and explore the outcomes and experiences using large-scale studies in different contexts associated with this patient-centric virtual care solution for CKD patients receiving PD at home.

## Abbreviations

CKD: chronic kidney disease
ESRD: end-stage renal disease
LHIN: Local Health Integrated Network
PD: peritoneal dialysis
RCT: randomized controlled trial
RE-AIM: Reach, Effectiveness, Adoption, Implementation, and Maintenance
